# Rebooting Regulatory T Cell and Dendritic Cell Function in Immune-Mediated Inflammatory Diseases: Biomarker and Therapy Discovery under a Multi-Omics Lens

**DOI:** 10.3390/biomedicines10092140

**Published:** 2022-08-31

**Authors:** Dimitra Kerdidani, Nikos E. Papaioannou, Evangelia Nakou, Themis Alissafi

**Affiliations:** 1 Immune Regulation Laboratory, Center of Basic Research, Biomedical Research Foundation Academy of Athens, 11527 Athens, Greece; 2 Laboratory of Biology, Medical School, National and Kapodistrian University of Athens, 12462 Athens, Greece

**Keywords:** immune-mediated inflammatory disorders, autoimmune diseases, immune regulation, dendritic cells, regulatory T cells, omics, therapeutic targeting, biomarkers, chronic inflammation

## Abstract

Immune-mediated inflammatory diseases (IMIDs) are a group of autoimmune and chronic inflammatory disorders with constantly increasing prevalence in the modern world. The vast majority of IMIDs develop as a consequence of complex mechanisms dependent on genetic, epigenetic, molecular, cellular, and environmental elements, that lead to defects in immune regulatory guardians of tolerance, such as dendritic (DCs) and regulatory T (Tregs) cells. As a result of this dysfunction, immune tolerance collapses and pathogenesis emerges. Deeper understanding of such disease driving mechanisms remains a major challenge for the prevention of inflammatory disorders. The recent renaissance in high throughput technologies has enabled the increase in the amount of data collected through multiple omics layers, while additionally narrowing the resolution down to the single cell level. In light of the aforementioned, this review focuses on DCs and Tregs and discusses how multi-omics approaches can be harnessed to create robust cell-based IMID biomarkers in hope of leading to more efficient and patient-tailored therapeutic interventions.

## 1. Introduction

Immune-mediated inflammatory diseases (IMIDs) are a diverse group of incurable clinical disorders that constitute a unique conceptual and medical challenge for the scientific community. Under the umbrella of the broad term IMIDs, many autoimmune as well as chronic inflammatory diseases, such as rheumatoid arthritis (RA), inflammatory bowel disease (IBD), systemic lupus erythematosus (SLE), type 1 diabetes (T1D), cutaneous inflammatory disorders (including psoriasis and atopic dermatitis (AD)), asthma and autoimmune neurological diseases such as multiple sclerosis (MS), can be incorporated. IMIDs develop as a consequence of complex mechanisms that depend on genetic, epigenetic, molecular, cellular, and environmental elements and result in defects in immune regulatory checkpoints of tolerance [1,2]. This breakdown of self-tolerance leads to the aberrant activation of lymphocytes against otherwise harmless self or foreign antigens causing chronic unrestrained inflammation that destroys self-organs and tissues.

Two key checkpoints of self-tolerance and decision-makers of the type and magnitude of the immune response are dendritic (DC) and regulatory T (Tregs) cells. On the one side, DCs, by up-taking environmental cues, self or foreign antigens and translating them into signals for the proper initiation of the immune response, constitute the sensors of the immune system and the link between innate and adaptive immunity [3,4]. On the other side are Tregs, that respond to signals of DCs, regulating and restraining exacerbated inflammation, thus comprising the brakes of the immune response [5,6]. During IMIDs, both cell types have been reported to be dysregulated, with altered frequencies in the periphery of patients, overt activation, and certain degrees of imbalance in their phenotype and function [7,8,9,10], thus leading to the breakdown of self-tolerance. Although the previous two decades have been transformative for the understanding of the mechanisms that govern immune dysregulation in IMIDs, effective and highly targeted treatments have proven to be elusive. Evidently, IMIDs remain a major burden on health systems around the world, accounting annually for several billion EUR in medical costs and lost income. Deciphering in depth the cellular and molecular mechanisms that contribute to the breakdown of immune tolerance is thus an important goal, with the prospect that this knowledge will pave the way to new clinical advances in the treatment of IMIDs.

The recent breakthrough in advanced multi-omics technologies provides the essential tools to ease the massive and in-depth understanding of the mechanisms driving immune dysfunction in IMIDs. Indeed, bulk and single-cell omics, multi-parameter flow and mass cytometry, next-generation spatial omics, and systems biology are among the current approaches expected to be applied in daily clinical practice for the upgrade of patients’ management and quality of life. Here, we focus on Tregs and DCs, the two fundamental gatekeepers of the immune tolerance and discuss how recent advances in the field of IMIDs, illuminated by the dawn of omics technologies, can be harnessed to create robust cell-based biomarkers and patient-tailored therapeutic interventions.

## 2. Regulatory T Cells as Multifaceted Orchestrators of Immune Responses

Tregs are an important immune system component, critical for maintaining homeostasis and immunological self-tolerance [11,12]. Tregs exert their suppressive functions either by cell-to-cell contact or secretion of cytokines. More specifically, Tregs can effectively suppress immune responses via (a) secretion of anti-inflammatory cytokines such as interleukin-10 (IL-10), IL-35, and TGF-β [13,14,15,16], (b) granzyme and perforin-mediated cytolysis [17,18,19], (c) expression of nucleotide-metabolizing enzymes such as CD39 and CD73 [20,21,22], (d) competing with effector T cells for IL-2, an essential T cell survival cytokine [23,24], and (e) dampening the maturation/antigen-presenting capacity of dendritic cells [25,26,27].

Both human and murine Tregs are phenotypically distinguishable by the expression of the transcription factor Foxp3 and the IL-2 receptor alpha chain (IL-2Rα, CD25). However, since CD25 can also be highly expressed in other subsets of activated CD4^+^ T cells in humans, the absence of the IL-7 receptor alpha chain (IL-7Rα, CD127) is complementarily used to identify human Tregs [28]. Expression of the master regulator Foxp3 is a cardinal feature of Tregs, fundamental for their development and suppressive function [6,29]. Therefore, loss-of-function mutations of the *FOXP3* gene in humans lead to the development of a severe autoimmune disease termed immune dysregulation, polyendocrinopathy, enteropathy, and X-linked (IPEX) syndrome [30,31]. Miyara et al. classified CD4^+^Foxp3^+^ Tregs from the peripheral blood of healthy individuals into three main fractions: Fr.I naïve Tregs (CD45RA^+^/CD25^low^), Fr. II effector Tregs (CD45RA^−^/CD25^high^) and Fr. III not Tregs (CD45RA^−^/CD25^low^) [32]. This classification, which is based on surface markers, nicely correlates to Tregs’ epigenetic profile and suppressive function with Fr. I and II being suppressive resting or activated Tregs, respectively, and Fr. III being non-suppressive and cytokine secreting non-Tregs [8]. 

Tregs are either generated in the thymus (thymic-derived Tregs, tTreg) or in the periphery through conversion of CD4^+^Foxp3^−^ T conventional cells following antigenic stimulation in the presence of TGF-β and IL-2 (induced Treg, iTreg) [33,34]. Whereas Treg cells were traditionally considered a terminally differentiated population, it is now well accepted that they acquire plasticity that allows them to adapt to the cues of the microenvironment [8]. By acquiring expression of specific lineage T cell-transcription factors, such as T-bet, GATA-3, IRF-4, STAT-3, RORγt, Bcl-6, and chemokine receptors, Tregs can skew to Th1, Th2, Th17, or T follicular helper cell-like phenotypes [35,36,37,38,39,40,41]. These functional adaptability is context and tissue-dependent [8].

Th1-like Tregs circulate in the blood of patients with autoimmune diseases [9]. Except for T-bet, they also upregulate CCR5, CXCR3, and secrete IFN-γ, while displaying reduced suppressive capacity when compared to Tregs. IFN-γ secretion has been shown in vitro to depend on PI3K/AKT/FoxO signaling [9,42]. Respectively, Th2-like Tregs upregulate GATA-3 and IRF-4 and secrete IL-4 and IL-13 [9]. In the setting of IMIDs, Th2-like Tregs have been found in tissues rather than the periphery [43]. Th17-like Tregs upregulate the transcription factor RORγt and secrete IL-17A. Although it is yet unclear whether they are a stable subcluster of Tregs or a transitory stage of Tregs to Th17 cells, Th17-like Tregs are found in steady-state in the gastrointestinal tract, where they have a protective role [40,44], but also in the synovium of arthritic patients and in psoriatic lesions where they contribute to disease pathogenesis [9,45,46,47].

Adding up to their heterogeneity, Tregs possess also a certain degree of instability. Unstable Tregs, named ex-Tregs, produce inflammatory cytokines, downregulate Foxp3 expression, and concomitantly lose their suppressive function [46,48,49]. Post-translational modifications of the Foxp3 protein, namely acetylation, phosphorylation, and ubiquitination of specific residues also contribute to Tregs’ instability and plasticity as they may lead to Foxp3 protein stabilization or proteasomal degradation [50,51,52]. Tregs’ instability seems to have a key role in the pathogenesis of autoimmune diseases [49]. However, the extent to which both Treg plasticity and instability contribute to the pathogenesis of IMIDs and whether the modulation of Tregs’ state can be proven therapeutically relevant is under investigation. 

Lastly, major advances in the field have uncovered Tregs that reside in non-lymphoid structures and contribute to tissue homeostasis rather than immune surveillance [53]. Tissue-resident Tregs have been identified in several tissues including adipose tissue, skin, lung and gastrointestinal tract where they become epigenetically adapted to microenvironment’s specific cues [54]. Thus, the transcriptomic profile of tissue-resident Tregs varies significantly with that of their lymphoid tissue counterparts, as well as among different tissues. Several markers have been identified that distinguish tissue-Treg precursors that reside in lymphoid organs prior to their transport to homing tissues, such as PPARγ^low^, TCF1^low^, ID3^low^, and NFIL3^+^ [55]. Nevertheless, tissue-resident Treg biology remains largely unexplored and constitutes a fruitful field of research.

Tregs are instrumental in preventing IMIDs and preserving immune homeostasis. In fact, most autoimmune diseases bear numerical or functional alterations in their Treg cell compartment. For example, in T1D, the activated Tregs (CD4⁺CD45RA⁻Foxp3^high^) in peripheral blood of patients are increased in numbers and functionally impaired akin to a pro-inflammatory phenotype [56,57,58]. In RA patients, although the frequencies of Tregs (CD4^+^CD25^+^CD127^−^) in the periphery are either similar or lower compared to healthy controls [32,59,60], Tregs in the synovial fluid are increased in numbers and less suppressive [32,61]. Individuals suffering from relapsing-remitting MS, in most of the studies, have decreased numbers of CD4^+^CD25^+^ Tregs and increased frequencies of Th1-like (CD4^+^CD25^high^CD45RA^−^CD127^−^Foxp3^+^) Tregs in their blood [32,59,60,62,63]. The latter have been shown to express the pro-inflammatory cytokine IFN-γ and have reduced suppressive function when co-cultured with effector T cells in vitro [61]. In line with the perturbed function and frequency of Tregs noted in various autoimmune diseases, Tregs (CD4^+^CD25^high^) numbers are also decreased in the peripheral blood of SLE patients and demonstrate reduced suppressive capacity relative to healthy controls [32,60,64]. Although human studies that investigate Treg frequencies and function in various autoimmune diseases suffer from discrepancies due to a lack of consistency in Treg definition markers, they nevertheless reveal the significance of Tregs for immune homeostasis [59].

Studies of Tregs in IMIDs derive mostly from data acquisition of flow cytometry and ex vivo assays, thus lacking collective and high-throughput insight. Recent technological advances have established multi-omics platforms in the field of research and offer holistic approaches to data acquisition that are unbiased and hypothesis-driven independent. Herein, we review Treg-specific multi-omics approaches that have been applied in IMIDs research up to date.

### 2.1. Transcriptomic Studies Paving the Way for Illuminating Tregs’ Functional Profiles and Subsets in IMIDs

The study of bulk mRNA transcripts within a biological sample, termed transcriptomics, has now become a standard approach for investigating molecular mechanisms that underlie steady-state and pathogenic conditions, as transcriptional profiling of cells is able to reveal gene function and gene structure [65]. By moving onward to the single-cell era it became apparent that transcriptomics at the single-cell level have reshaped modern research and have uncovered cellular differences and the heterogeneity of biological samples that have been masked by bulk RNA-sequencing (RNA-seq). Mostly bulk, and to a lesser extent single-cell RNA-sequencing (scRNA-seq), have been applied thus far in studying Tregs in the context of IMIDs.

A recent study in our lab interrogated the transcriptomic profile of Tregs from the peripheral blood of individuals suffering from MS, RA and SLE [60]. RNA-seq analysis revealed a plethora of deregulated transcripts when compared to healthy controls. Tregs were predominately altered in metabolic pathways related to oxidative stress, mitochondrial dysfunction, cell death and DNA damage response. Interestingly, this signature was consistent across all autoimmune disease settings studied [60].

As mentioned above, Tregs are able to adapt to specific microenvironments, thus conditions such as excessive inflammation imprint onto Treg profile. It has been reported by two independent studies that in juvenile idiopathic arthritis (JIA), Tregs obtained from inflamed joints have a specific effector profile [66,67]. Both studies compared, among others, the transcriptome of Tregs from synovial fluid to those of peripheral blood of individuals with JIA. Differential gene expression analysis revealed that Tregs in the synovial fluid express a Th1 transcriptomic signature that is characterized by the expression of transcription factor *TBX21* (T-bet), chemokine receptor *CXCR3*, and IL-12 receptor β2 (*IL12RB2*). IFN-γ was also found upregulated in one of the studies [66], nevertheless, when Tregs were stimulated ex vivo they failed to produce this cytokine [67]. Despite high expression of Th1-related proteins, Tregs preserved their suppressive features as shown by the maintenance of a robust Treg-associated transcriptional program [66] and functional assays [66,67].

Julé et al. employed scRNA-seq on Tregs sorted from synovial fluid of individuals experiencing JIA. Among five Treg clusters identified in this study, cluster 1 matched the expression profile of Th1-like Tregs while preserving the Treg transcriptomic signature, thus confirming the uncovering of a stable effector Treg population that maintains Treg-specific demethylation patterns and suppressive capacity, as identified by bulk RNA-seq. The newly identified and highly suppressive population of Th1-like Tregs, which was unveiled through the prism of transcriptomics, could constitute an attractive target with important therapeutic benefits for individuals with JIA. Four additional Tregs subpopulations were identified that spanned from the classical and highly activated HLA-DR^+^ Tregs that robustly express Treg signature genes to the CD161^+^ and IFN-induced Tregs that share some genes with effector T cell clusters [66].

Recently, the transcriptome of Tregs in the blood of individuals experiencing autoimmune polyendocrine syndrome type I (APS-1) versus healthy controls has been interrogated [68]. Whereas only subtle changes were observed between disease and healthy groups, the G Protein-Coupled Receptor 15 (*GPR15*) gene was found significantly downregulated, while the Fatty Acid Synthase (*FASN*) gene was upregulated in APS-1 Tregs [68]. Given that individuals with APS-1 suffer from gastrointestinal manifestations and GPR15 is a homing receptor for the gut, it was speculated that GPR15 downregulation might be indicative of a defective influx of Tregs in the gut [68]. In addition, an increase in FASN, important for fatty acid synthesis, is suggestive of metabolic reprogramming of APS-1 Tregs [68]. However, data were strictly descriptive and deprived of functional evidence, thus results must be considered cautiously.

Despite their dominant role in immunosuppression, so far only a very limited number of studies have focused on the single-cell analysis of tissue-specific Treg cells in IMIDs. In one of them, scRNA-seq was used to characterize Treg cells isolated from the peripheral blood and synovial fluid of two individuals with ankylosing spondylitis (AS) [69]. Analysis revealed ten specialized Treg clusters, present in both tissues, with unique gene expression signatures. Among them, a CD8^+^ Treg subset expressing cytotoxic markers such as granzyme B and granulysin was significantly enriched in the synovial fluid of individuals with AS, whereas a Th17-like RORC^+^KLRB1^+^ Treg subset characterized by IL-10 and LAG-3 expression was significantly enriched in the blood of AS patients. Despite the small size of samples, these two clusters were also identified in the peripheral blood and synovial fluid of individuals with psoriatic arthritis, another type of spondyloarthritis (SpA) [69]. Total synovial fluid Tregs were characterized by the upregulation of activation and inhibitory markers, as well as TNF and interferon response genes, and they were clonally expanded suggesting tissue-specific adaptation. Thus, targeting these unique characteristics of joint-specific Treg subsets could have promising applications for the amelioration of SpA.

Immune-related adverse events (irAEs) are an atypical IMID that is worth mentioning. The impressive success of immune checkpoint therapies in the treatment of various types of cancer is often overshadowed by irAEs that arise due to excessive activation of the immune system. Previous studies in our lab applying RNA-seq have demonstrated that Tregs from the peripheral blood of individuals developing irAEs bear a pro-inflammatory profile accompanied by enrichment in the apoptotic and metabolic pathways [70]. Moreover, irAEs-Treg signature is shared across different types of cancer and resembles Treg traits of individuals with autoimmune diseases [70]. Unraveling phenotypic switches of Tregs that drive or precondition the development of irAEs is of utmost importance for the prevention of toxicities that often accompany cancer immunotherapies.

Although not an IMID per se, graft-versus-host disease (GVHD) manifests as an autoimmune disease, and transcriptomic approaches have been employed to dissect Treg complexity in patients receiving hematopoietic stem cell transplantation [71]. Specifically, single-cell transcriptomic analysis was performed in Tregs of the peripheral blood and bone marrow of healthy donors and patients after hematopoietic stem cell transplantation that were either experiencing GVHD or not. The analysis resolved nine clusters both in the peripheral blood and bone marrow of individuals that included naïve (CCR7^hi^), activated (HLA-DR^hi^), LIMS1^hi^, effector (Foxp3^hi^), and proliferative (MKI67^hi^) Tregs. Functional evaluation revealed MKI67^hi^ and Foxp3^hi^ clusters as highly suppressive, followed by HLA-DR^hi^ and LIMS1^hi^ clusters. Pseudotime trajectory analysis uncovered the transition among clusters according to which naïve Tregs followed two distinct differentiation pathways towards either Foxp3^hi^ Tregs (Path 1) or MKI67^hi^ Tregs (Path2). Whereas similar clusters, spanning from naïve to activated/effector Tregs, were identified in all groups, effector Tregs clusters in individuals developing GVHD displayed downregulation of suppression and migration pathways as well as a senescence-like signature compared with non-GVHD patients [71]. Although the latter can be attributed to the age gap between GVHD and non-GVHD patients, Treg interrogation on a single-cell level offered a greater understanding of Treg features upon GVHD.

Regarding organ-specific immune-mediated diseases, the role of cell-based omics technologies, and particularly the advances in single-cell TCRαβ sequencing, is of primary importance to illuminate the antigen specificities of the pathogenic cells that mediate tissue damage, or of the regulatory cells that suppress the former in the inflammatory niche. Such knowledge will be decisive during the design of more efficient and targeted therapeutic approaches such as autoantigen-specific TCR engineering.

One such case is T1D in which Treg cells have already been exploited in therapies, with early phase clinical trials of ex vivo expanded polyclonal Treg cells showing promising results [72,73]. However, since polyclonal Tregs are not antigen-specific, the approach utilized in these clinical trials could potentially lead to systemic unwanted immunosuppression. Interestingly, preclinical studies using the non-obese diabetic (NOD) murine model for T1D revealed that relatively small numbers of antigen-specific Treg cells, either pancreatic lymph node-derived or genetically engineered, and not polyclonal Treg cells, could prevent and even reverse T1D, pointing to therapies utilizing diabetogenic TCR-expressing Treg cells [74,75]. Still, most antigen-specific Treg cells are tissue-resident and only a small portion of them circulates in the bloodstream, rendering them difficult to isolate and characterize in humans. Additionally, so far, the attempts to create tailored Tregs utilize recombinant TCRs from Teff cells [76,77]. Due to these challenges, up to now the identification of the exact TCR sequences specific for dominant diabetogenic epitopes in Treg cells has been restricted only to NOD mice. To this end, Spence et al. employed TCR repertoire profiling and TCRαβ scRNA-seq to determine the specificity of Treg cells in the islets of Langerhans. Treg clonotypes were found to be expanded and the least diverse in inflamed islets compared to other lymphoid organs, while some of their TCRs were specific for islet-derived antigens including insulin B:9–23 and proinsulin, implying tissue-specific antigen-driven expansion of Treg clonotypes [78]. Their transcriptomic observation was further confirmed utilizing insulin B:9-23 tetramers able to detect increased insulin-specific Treg clones in the islets of NOD mice. Moreover, the adoptive transfer of total Treg cells from the islets, but not of Tregs from lymphoid organs, in NOD.CD28^−/−^ mice could lead to disease rescue, further supporting the suitability of engineered Treg cells expressing insulin-specific TCRs as a promising strategy for suppressing autoimmune reactions against beta cells.

In JIA, TCR repertoire assessment on a single-cell level revealed that the Th1-like Tregs identified in the joints of individuals with JIA are bone fide Tregs, as their clonotypic composition was similar to that of other Treg clusters and not to effector T cells [66]. Another study has identified a subpopulation of activated Tregs (HLA-DR^+^) in the blood of JIA and RA patients that has been negatively correlated to response to therapy [79]. In JIA, the so-called inflammation associated (ia) Tregs expand during inflammation and decrease when the disease is inactive. It is important to note that iaTregs also expand when children have poor responsiveness to therapy. TCR-seq revealed antigenic stimulation and shared clonotypes between these iaTregs and Tregs from the synovium [79]. This observation confirmed the fact that HLA-DR^+^ Tregs recirculate between the synovium and blood, which could only be hypothesized up to then by the expression of tissue-homing receptors [79]. Migrating to blood-synovial Tregs could offer easy, non-invasive access to arthritis-associated clonotypes and at the same time could be exploited to monitor response to therapy [79].

### 2.2. Unraveling the Epigenetic Mechanisms Governing Tregs Links Molecular Traits to Pathogenicity

Marking the epigenetic changes across many genes is another available multi-omic tool termed epigenomics. Gene expression is driven by promoters, enhancers, insulators, etc. Epigenetic regulation of enhancers via histone modifications, which reveals gene regulation, has been used in IMIDs research [80]. Epigenomic approaches often act conjointly with transcriptomics to uncover context-specific gene regulation, as changes noted at the mRNA level are sought to be reflected also at the epigenetic level [67]. ChIP-seq was performed to profile histone modification marks that indicate transcriptionally active enhancers (acetylation of lysine 27 on histone H3 (H3K27ac) and monomethylation of lysine 4 on histone (H3K4me1)) using Tregs obtained from the synovial fluid versus peripheral blood of individuals with JIA. The study validated the Th1-like profile of synovial fluid-Tregs that was observed from RNA-seq data [66,67]. Specifically, ChIP-seq identified super-enhancers of genes that were found upregulated in mRNA levels such as *TBX21* and *IL12RB2* as well as super-enhancers associated with putative Treg markers, indicating that Tregs in the inflammatory environment of arthritic joints are adapted to a Th1-related profile while maintaining Treg specific features [67]. The same study uncovered vitamin D receptor (VDR) as one of the top predicted regulators of Treg differentiation in the arthritic joints, marking it as an attractive therapeutic target. Ex vivo stimulation with vitamin D3 skewed Tregs towards an effector Treg profile [67].

Similar epigenetic profiling was performed in peripheral blood-Tregs in individuals with T1D versus healthy controls [81]. ChIP-seq and subsequent sophisticated in silico analysis revealed that (a) T1D-Tregs have fewer active enhancers compared to healthy Tregs, many of which regulate genes implicated in T1D pathogenesis, and (b) certain single nucleotide polymorphisms (SNPs) in enhancer regions disrupt the binding of key transcription factors that regulate transcriptome changes in T1D-Tregs [81]. Similar studies that translate, via multi-omics approaches, non-coding genetic variants to functional/pathological states of Tregs are needed for the prediction and understanding of IMIDs.

ChIP-seq along with ATAC-seq that determines chromatin accessibility and RNA-seq have also been used to highlight Tregs’ contribution to the development of IMIDs at large [82,83,84,85,86]. Epigenetic profiling of Tregs from the peripheral blood of healthy individuals revealed that autoimmune disease-associated SNPs are enriched in hypomethylated regions of naïve Tregs that control transcription and epigenetic changes, hence Treg function [86]. A recent study further supports the functional relevance of SNPs by showing that immune disease variants reside in chromosomal loci involved in Treg cell activation and IL-2 signaling [83]. In general, genetic variants associated with immune diseases are found enriched in regulatory regions of Tregs [82,84,85]. Multi-omics approaches combined with genome-wide association studies (GWAS) pave the way for the understanding of Tregs’ contribution to IMIDs and the discovery of new Treg-specific therapeutic targets.

### 2.3. Proteomic Studies Shed Light on Distinct Treg Subsets with Opposing Functions

Proteomic analyses have helped us elucidate the mechanisms of inflammation-mediated pathology. They have also long been considered a valuable platform for the identification of autoimmune disease biomarkers for diagnostic and prognostic purposes in accessible biological fluids. In the new multi-omics era, approaches combining cell-type-based proteomics with transcriptomics could foster the characterization of disease-specific Treg subtypes which may serve as biomarkers for disease initiation or progression. However, up to date, only one study has focused on the proteomic profiling of Tregs in the context of IMIDs.

In this study, Weerakoon et al. employed proteomics in sorted Tregs (CD4^+^CD25^high^CD127^−^) and conventional CD4^+^ T cells (CD4^+^CD25^−^) from the peripheral blood of IBD patients. Their analysis pinpoints the absence or presence of integrin CD49f as a marker that distinguishes conventional T cells from Tregs. However, among Tregs, CD49f expression was also variable, and could separate two Treg subsets with distinct functions in the peripheral blood of IBD patients. CD49f ^−^ Tregs show increased suppressive ability and expression of inhibitory receptors, whereas CD49f^high^ Tregs possess a proinflammatory phenotype and they are increased in the blood of IBD patients with active disease. They also suggest that the ratio CD49f^high^/CD49f ^−^ Tregs may constitute a useful predictor of disease activity, but this result should be validated in larger cohorts of patients [87]. Still, it is beyond doubt that more studies in the field of Treg proteomics in IMIDs are needed in order to disentangle the protein profile of these cells and identify novel Treg-specific markers and potential therapeutic targets. Furthermore, following the road paved by single-cell transcriptomics, newly developed single-cell proteomic platforms have the potential to uncover additional layers of Tregs’ complexity in the setting of IMIDs.

### 2.4. Microbiome-16S-Sequencing at the Crossroads between Tregs and Microbiota, Leading the Way to Microbiota-Related Therapeutic Interventions

Commensal microbes colonize barrier sites where they are essential for immune homeostasis predominantly by modulating the generation of Treg cells. The advancements in 16S rRNA and metagenomics sequencing technologies have shed light on the composition and function of the human microbiome, as well as its direct role in modulating immune responses through its components or metabolites [88]. Increasing evidence suggests that gut dysbiosis is implicated in many IMIDs including SLE [89,90], RA [91,92], IBD [93,94], T1D [95], Grave’s disease [96] and MS [97,98], and it is characterized by a reduction in small-chain fatty acid (SCFA)-producing species. Given the importance of Treg cells in establishing immune tolerance to self-antigens and commensal microbes, researchers’ attention is now shifted towards Treg–microbiota interactions in autoimmune disorders, which may underpin the decreased numbers and/or dysfunction of Tregs in these conditions. Specifically, it has been shown that in mice, the SCFA butyrate promotes the induction of Treg cells, whereas treatment of naïve T cells with butyrate-enhanced histone 3 acetylation in the promoter and conserved non-coding sequence regions of the *FOXP3* locus leads to differentiation into Treg cells [99]. These unique effects of butyrate on Treg cells could provide protection from diabetes in NOD mice fed with a diet that generates large amounts of butyrate after colonic fermentation [100].

Another study showed that microbial species found in fecal samples of SLE patients induced a pro-inflammatory immune phenotype characterized by lymphocyte activation and Th17 differentiation. Interestingly, supplementation of SLE stool samples with Treg-inducing bacteria could restore Treg/Th17/Th1 imbalance [90]. Furthermore, long-term propionic acid supplementation in MS patients could reduce the annual relapse rate and ameliorate disease progression by increasing Treg cell numbers and suppressive function [98]. Thus, further exploring the crosstalk between Tregs and microbiota by integrating information from different high-throughput technologies (single-cell, metabolomics) will facilitate the development of therapeutic interventions that restore immunological tolerance through manipulation of the microbiome.

**Figure 1:**
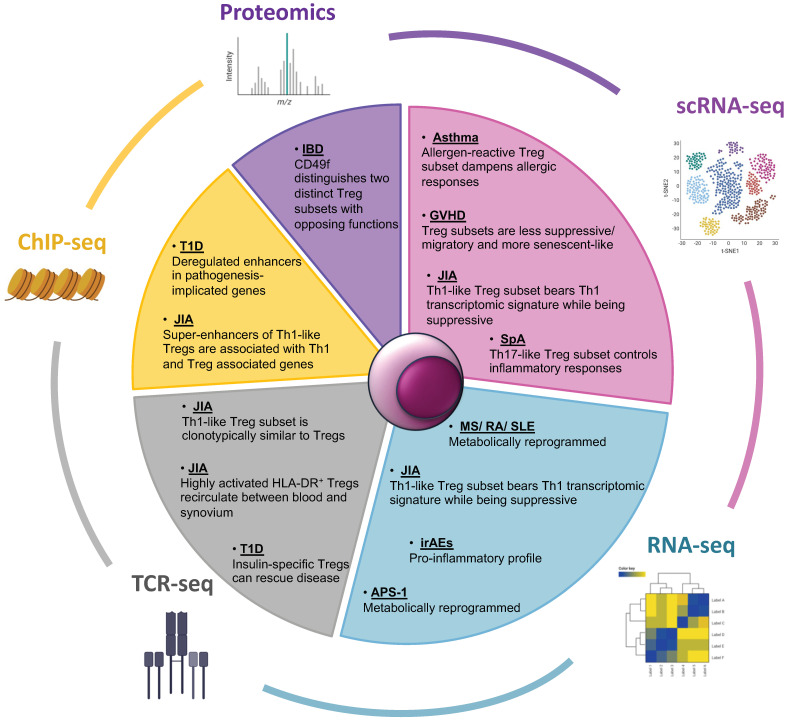
Multi-omics approaches utilized in IMIDs research, focusing on regulatory T cells. The Pie chart depicts omics technologies that have been used to study the contribution of regulatory T cells in the pathology of IMIDs. Predominantly RNA-seq but also proteomic and epigenomic technologies have revealed Treg profiles that are suppressive, pro-inflammatory, or metabolically reprogrammed, as well as distinct Treg subsets across various diseases. scRNA-seq, single cell RNA-seq; ChIP-seq, Chromatin Immunoprecipitation sequencing; TCR-seq, T Cell Receptor sequencing; GVHD, Graft Versus Host Disease; JIA, Juvenile Rheumatoid Arthritis; SpA, Spondyloarthritis; MS, Multiple Sclerosis; RA, Rheumatoid Arthritis; SLE, Systemic Lupus Erythematosus; irAEs, immune related Adverse Events; APS-1, Autoimmune Polyendocrine Syndrome Type I; T1D, Type I Diabetes; IBD, Inflammatory Bowel Disease.

Key observations by studies employing transcriptomic, proteomic and epigenomic approaches have provided insight into Treg cells’ function and contribution to the pathogenesis of numerous IMIDs (Figure 1). In the single-cell era, multi-omics approaches are indispensable for understanding the perplexing mechanisms that underlie Treg cell biology in IMIDs, the elucidation of which can lead to specific and effective therapeutic regimes.

## 3. Dendritic Cells as Multifaceted Orchestrators of Immune Responses

Ever since their initial discovery by Steinmann and Cohn [101], DCs have grown from simply being viewed as highly motile stellate cells to being recognized as an essential connective link between the innate and adaptive arm of immunity in mammals. DCs constantly sample their microenvironment by engulfing self or non-self antigenic molecules. By possessing a large array of surface and intracellular receptors, they integrate the context in which these molecules are met and thus whether they are associated with invading pathogens, damaged cells or constitute innocuous antigens. After antigen processing, DCs present peptides to T cells, thereby activating them in an antigen-specific way. Most importantly, the induced T cell activation is polarized accordingly, through the production of cytokines and provision of specific costimulatory signals in order to ensure either sufficient protection against the pathogen met, or establishment and maintenance of tolerance against self and innocuous antigens [102,103,104]. This has earned them the title of orchestrators of immune responses.

The multifaceted role of DCs in immune responses is a derivative of their heterogeneity. Notably, the DC term functions as an umbrella that encloses several cell subsets, each possessing distinct developmental requirements, phenotype and functional properties [102,105]. While DCs have initially been studied more extensively in mice, with the help of multi-omics approaches, recent publications have elegantly dissected the human DC compartment, elucidating in parallel a high interspecies conservation of their development, phenotype and function [105,106,107]. Among DCs, two main distinct lineages can be distinguished, namely conventional DCs (cDCs) and plasmacytoid DCs (pDCs).

In both mice and humans, pDCs have a prominent role in anti-viral defense due to their ability to secrete copious amounts of type I interferons (IFN) in response to virally derived nucleic acids [108]. The efficiency of pDCs in antigen presentation and T cell activation is still not clearly defined due to controversial findings between different experimental settings [109,110,111,112]. While their exact developmental trajectory has also been a highly debated topic in recent years, [113,114,115] the consensus is that their differentiation is dependent on the transcription factor E2-2 in both species [107,108]. On the contrary, their major defining phenotypic markers seem to be not so well-conserved. Despite MHC-II/HLA-DR expression being a common trait, murine pDCs are characterized as B220^+^, SiglecH^+^, CD317^+^, Ly6C^+^, CD11c^intermediate^,while in humans characteristic pDC markers are CD123, CD303, CD304, combined with a lack of CD11c and CD5 expression [107,108,116].

cDCs excel in the activation of adaptive immune responses by presenting antigens to T cells [105]. They are subsequently divided into cDC1 and cDC2 and exhibit a remarkable division of labor when it comes to their role in immune responses [105]. Both cDC subsets are characterized by the expression of CD11c and MHC-II/HLA-DR but are distinct in dependence on transcription factors and the expression of other surface markers. Continuous and high expression of the transcription factors IRF8 and BATF3 is a prerequisite for maintaining the developmental and functional program of both human and murine cDC1 [106,117,118,119]. Genetic approaches have additionally elucidated the role of ID2 [120] and NFIL3 [121,122] in mouse cDC1 development, however, their implication in humans has yet to be determined. In terms of their phenotype, murine cDC1 can be reliably identified across tissues by the expression of XCR-1, CLEC9A, CD24 and CD205 [105]. Moreover, CD8α and CD103 are used as cDC1 characteristic markers in lymphoid and non-lymphoid tissues, respectively, despite the latter also being expressed in an intestinal cDC2 population [105]. In addition to XCR-1 and CLEC9A, human cDC1 in both blood and non-lymphoid tissues have characteristic expression of CD141 and CADM1 [107,116]. Functionally, cDC1 play a dominant role in inducing cytotoxic CD8^+^ T and Th1 polarized CD4^+^ T cell responses against intracellular pathogens, such as viruses and bacteria, but also participate majorly in antitumor immunity [105]. They do so via producing ample amounts of IL-12 that activates T cells both directly and indirectly by promoting a Th1-favorable cytokine milieu from bystander cells [119,123,124]. Added to the above, their remarkable potential as CD8^+^ T cell activators is extended by their ability to cross-present extracellular antigens on MHC-I molecules [119,123,124]. In contrast to the pro-inflammatory role described above, especially in mice, the high potential of cDC1 to induce peripheral regulatory T cells has also been proposed [125,126].

In contrast to pDCs and cDC1 subtypes, the phenotype and developmental requirements of cDC2 between humans and mice seem to overlap the least. In mice, studies have identified transcription factors IRF-4, ZEB2, KLF4 and RELB as central mediators of cDC2 development [102,105,122] as well as pathways with more tissue-specific context such as NOTCH and retinoic acid signaling [127]. While human cDC2 distinctively expresses IRF-4, its role in their development is not yet elucidated. Characteristic murine cDC2 surface markers include CD11b, CD172a, CD4 and CLEC4A4 [105] of which only CD172a is a common defining marker with their human counterparts. The latter are additionally identified by their expression of CD1c, FcεR1α and CLEC10A [107,116]. Functionally, human and murine cDC2 align and are believed to be more efficient in inducing CD4^+^ T cell activation and polarization towards Tfh, Th2 or Th17 effector responses, crucial for T cell-dependent antibody production by B cells, defense against multicellular pathogens such as helminths or extracellular bacteria and fungi, respectively [128,129,130,131,132,133,134,135]. Their CD4^+^ T cell activation pattern also extends to regulatory directions via the induction of Tregs both in the thymus and in peripheral tissues [136,137]. Remarkably, cDC2 have been found to exhibit the highest intra-subset diversity compared to pDCs and cDC1. This heterogeneity, despite being ever-growing, has been studied in detail in mice [138,139,140], however, it has only recently been appreciated in humans.

In addition to pDCs and cDCs, a new cell subset termed transitional DCs has quite recently been identified in both humans and mice [141]. As implied by their name, these cells are placed in between the two aforementioned populations in the DC spectrum and have been described to possess shared pDC and cDC properties. Nonetheless, their exact function is yet to be defined and needs to be investigated further.

Many studies focusing on DCs, and especially in humans, use peripheral blood monocytes as a source to generate them in vitro. While not ontogenetically related to pDCs and cDCs, these monocyte-derived DCs (moDCs) have been used extensively due to the existence of established protocols for their generation, the enhanced availability of monocytes in the peripheral blood and their implementation in clinical practice [107,142]. Similar protocols exist in mice, however, bone marrow rather than peripheral blood is the selected source to generate such cells [107]. Data suggest that mature in vitro-differentiated moDCs likely align with monocyte-derived cells arising under inflammatory conditions in vivo [107,143]. The latter cells are characterized by the expression of CD11c, MHC-II/HLA-DR, CD14, CD64, CD11b, CCR2, CD209 and CD206 in mice and humans with Ly6C positivity being an extra distinctive phenotypic trait of the murine cells [143]. In vivo generated moDCs have a profound pro-inflammatory potential and functional specialization, primarily connected with direct anti-microbial effector function, evident by the fact that there were first described in mice infected with *L. monocytogenes* [144]. Their T cell activation potential in most cases does not match that of cDCs, however, it is not redundant for the clearance of some pathogens requiring strong Th1 immunity [145]. As expected, their pro-inflammatory role can function as a double-edged sword, since these cells have been postulated to enhance many IMIDs manifestations [107,143].

### 3.1. Elucidating the Role of Dendritic Cells in IMIDs Utilizing Multi-Omics Approaches

**Figure 2:**
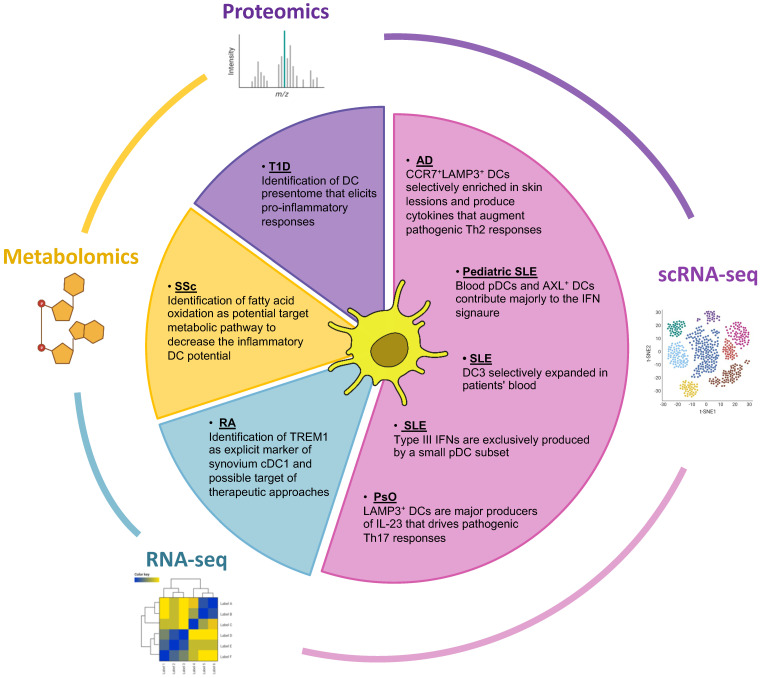
Multi-omics approaches utilized in IMIDs research focusing on dendritic cells. The Pie chart depicts omics technologies that have been used to study the contribution of dendritic cells in the pathology of IMIDs. Mainly scRNA-seq but also proteomic and metabolomic studies have highlighted dendritic cell subsets and inflammatory signatures that drive pathogenic responses in the disease spectrum of IMIDs. scRNA-seq, single cell RNA-sequencing; RA, Rheumatoid Arthritis; SLE, Systemic Lupus Erythematosus; T1D, Type I Diabetes; AD, Atopic Dermatitis; PsO, Psoriasis; Ssc, Systemic sclerosis.

Given their role in maintaining the balance between protective immune responses and self-tolerance, DCs play a critical part in IMID manifestations in which this balance is by default perturbed. Their detailed role has been extensively reviewed elsewhere [7], and in brief entails the dysregulation of one or more of the following functional properties: (a) perturbation in the pattern of secreted cytokines, quantitatively and qualitatively, that promote pro-inflammatory responses from other innate and adaptive immune system cells; (b) enhanced antigen presentation of primarily self-antigens; and (c) altered distribution in terms of both frequency and spatial arrangement, often related to differences in their migratory capacity, that affects especially the inflamed tissues but also peripheral blood. Here, we aim to report cases in which the role of DCs in IMIDs has been refined or enriched by the advent of recent omics approaches (Figure 2).

### 3.2. Bulk and Single-Cell RNA Sequencing Have Expanded the Portfolio of DC Subsets and Illuminated Their Role in IMIDs Perturbations

One remarkable advantage of multi-omics approaches is their potential for single-cell resolution. This was made apparent especially for human cDC2, as recent studies identified novel subsets within the CD1c^+^ cDC2 population using scRNA-seq coupled with index sorting [146,147]. The subdivision of these new subpopulations, namely DC2 (CD5^+/−^CD163^−^CD14^−^) and DC3 (CD5^−^CD163^+^CD14^+/−^), based on their immunophenotype was also found to be accompanied by functional differences [147,148]. In the context of IMIDs, CD163^+^ DC3s were found to be expanded in the blood of SLE patients and presented a highly activated phenotype compared to healthy controls. Interestingly, their frequency in blood was highly correlated to clinical scores. Secretome analysis showed that, among cDC2 subsets, DC3s uniquely produced many pro-inflammatory mediators when activated by the serum of SLE patients [147]. Given the above, it would be intriguing to investigate the performance of these cells as disease biomarkers and establish whether manipulating their function could ameliorate disease progression. Additionally, their role in other IMIDs such as RA and Psoriatic Arthritis (PsA) warrants further investigation due to their increased potential for induction of IL-17A producing T cells [147].

DC3s were also found selectively expanded, among cDC2, as assessed by scRNA-seq in pediatric SLE (cSLE) patients’ peripheral blood mononuclear cells (PBMCs), compared to age-matched healthy individuals [149]. Interestingly, overtaking even DC3s, the majorly expanded cDC cluster resembled the AXL^+^ DCs first identified by Villani et al. [146]. Additionally, although pDCs were found decreased as a total population in cSLE samples, further analysis revealed four distinct subclusters, one of which was profoundly expanded in SLE compared to healthy individuals. Notably, the defining markers of this expanded pDCs subcluster consisted primarily of interferon-induced genes, accompanied by genes connected to transcription factors (e.g., *STAT1, IRF7*) and antigen presentation (e.g., *CD74, HLA-DRA, CTSB*) [149]. The latter could point towards a yet unexplored role of these cells in propagating the IMID by activation of autoreactive T cells. In line with their initial placing on the verge between cDCs and pDCs, AXL^+^ DCs together with the expanded pDC subcluster were found to be among the PBMC clusters contributing the most to the SLE IFN signature. The above study proceeded a step further by aligning side by side the pediatric samples to corresponding samples from adults, highlighting age as another contributor to the fluctuation of disease-specific subclusters. Keeping up with the pDC and SLE field, Hjorton et al. investigated the cellular source of type III IFNs, a cytokine group whose contribution to the SLE IFN signature and disease progression remains poorly studied. To this end, they isolated pDCs from healthy donors, used a stimulation mix containing RNA immunocomplexes (used widely as IFN inducers in these cells) plus IFN2ab and IL-3, and subjected them to scRNA-seq [150]. Unexpectedly, they found that only a small population of single-cell sequenced pDCs contributed mostly to the total detected transcripts of both IFN III and I. Compared to the non-IFN III-producing cells, the identified small pDC cluster was also characterized by higher mRNA levels of genes connected to immune activation such as *TNF, CD40*, *CD83* and *IL12A*. While not explored by the authors, it would be interesting to speculate as to whether their identified pDC subcluster aligns with the expanded one mentioned by Belaid et al. [149], as in both cases its frequency among healthy donor pDCs was minimal. Thus, the importance of single-cell resolution in identifying the disease and age-relevant cell populations was once more signified.

In most cases, peripheral blood has been used as a mirror to study DC properties in IMIDs, however, analyses from inflamed tissues are equally or even more important as suggested by the expected effect of tissue microenvironments in DC transcriptional and functional signatures [151,152]. This site-specific analysis has been bolstered by recent omics advances, since their high throughput performance automatically decreases the required cell numbers to conduct meaningful experiments. As an example, Caravan and colleagues, studied the impact of the synovial microenvironment in cDCs from RA patients [153,154]. Using multiparameter flow cytometry and RNA sequencing, they found not only that cDCs were enriched in the synovial tissue of RA patients, compared to the blood of the same individuals as well as that of healthy controls, but that they also exhibited a highly activated phenotype as assessed by expression of costimulatory molecules [153,154]. Regarding the CD1c^+^ cDC2, the synovial microenvironment was shown to induce metabolic alterations, polarizing them to a more glycolytic phenotype while a more detailed analysis was performed for CD141^+^ cDC1. For the latter, the hypoxic synovium was shown to specifically induce the expression of TREM-1 as part of a site and disease-specific signature. Interestingly, in vitro crosslinking of TREM-1 in cDC1 isolated from synovial tissue could induce their activated phenotype in parallel to an increased ability to induce pro-inflammatory cytokine production from heterologous and autologous T cells [153]. Additionally, supernatants from these cDC1-T cell co-cultures could activate synovial fibroblasts to produce an array of soluble mediators consistent with the acquisition of an invasive phenotype. The authors concluded that the discovered synovium-specific signatures could be harnessed in order to design novel therapeutic and cDC targeted strategies, with TREM-1 being a frontline example.

Omics analysis targeted to the inflamed tissue is additionally essential for another IMID, namely AD. Two recent studies have attempted to interrogate the immune and non-immune skin compartments of patients with AD and healthy controls using scRNA-seq [155,156]. Both studies found that DC populations were expanded in the pathogenic samples in relation to healthy skin, with cDC2 probably being the more over-represented population due to their characteristic expression pattern of surface markers. Alongside the “typical” cDC populations, another smaller cluster expressing *CCR7* and *LAMP3* was identified. Despite their small numbers, these cells exhibited some very interesting traits such as clear characteristics of mature and migratory behavior and selective enrichment in the lesional skin of AD patients combined with their almost complete absence from healthy samples [155,156]. He et al. also found that these LAMP3^+^CCR7^+^ DCs robustly expressed type 2 chemokines such as *CCL17* and *CCL22*. These data were nicely corroborated by the fact that T cell populations with Th2 and Th22 polarization states were additionally enriched in the AD skin samples, opening the possibility that DCs are the major innate immune cell to attract these pathogenic T cells in the site of inflammation. Notably, these type 2 chemokines have already been used as reliable biomarkers to measure disease progression and response to therapy [157], however, the source cells were not clearly defined. Moreover, Rojahn et al. reported that added to type 2 chemokines, myeloid cells including DCs, produced amphiregulin in the lesional skin that can activate keratinocytes and thus worsen the clinical manifestations of AD [156]. Collectively, the above studies could be the starting point of further investigations on whether DCs are a major source of the above soluble factors and if so, implement their targeting as better therapeutic interventions and/or evaluation of them as more accurate biomarkers.

On the same page and similarly focusing on IMIDs with skin-related pathological manifestations, Kim and colleagues [158] interrogated the immune compartment of skin biopsies from patients with psoriasis as compared to healthy volunteers. To cope with the inherent issues introduced by enzymatic digestion of the skin as well as with the low leukocyte frequencies in that tissue, they implemented a novel approach by profiling with scRNA-seq the cells naturally emigrating from skin biopsies over the course of 48 h. In line with the studies above, they found DCs to be majorly expanded in the samples of psoriatic patients with a reported increase in their numbers over three-fold compared to healthy skin [158]. Interestingly, they identified DCs with both a mature and semi-mature phenotype. Semi-mature DCs, in both sample groups, were found to express genes encoding for IL-10 and CD141. While the authors did not elaborate further on this, the description could fit skin resident cDC1 and at the same time render these cells as potential targets of tolerance, re-establishing therapeutic approaches. Mature DCs, on the other hand, had higher expression of genes related to antigen presentation machinery and costimulatory signals, a signature that was further reinforced in psoriasis samples. A defining marker of these mature DCs was *LAMP3*, highlighting, in conjunction with the above studies in AD, that the same DC populations can have disease-promoting roles in a broad spectrum of IMIDs. Additionally, in psoriatic samples mature DCs expressed considerably more *IL-23A*, a cytokine related to the establishment of a pathogenic Th17 profile, while at the same time had markedly less expression of *KYNU*, an enzyme participating in the kynurenine pathway known for its immunomodulatory role. Going a step further, by using a computational algorithm to simulate cell-to-cell communication events, the authors were able to show that the increased *IL-23A* production by mature DCs in psoriatic skin would signal in *IL-17F* producing Th17 cells, shown to express the highest amount of the cognate receptor. Interestingly, these IL-17F^+^ cells were the largest subset of IL-17 producing T cells in psoriasis samples, therefore suggesting that their expansion and pathogenic function is a derivative of the pro-inflammatory secretory behavior of mature DCs.

### 3.3. Contribution of Proteomics in the Identification of DC-Presented Epitopes in IMIDs

As mentioned above, a prominent DC function lies in the processing and presentation of autoantigens to autoreactive T cells. However, an up-to-the-point question concerns whether specific epitopes dominate and are preferentially presented, even in cases that a particulate cell population is targeted. In the example of T1D, a recent study aimed to delineate the naturally processed and presented epitopes by DCs as derived from pancreatic beta cells [159]. The authors isolated peripheral blood CD14^+^ monocytes from healthy donors and cultured them in vitro with GM-CSF and IL-4 in order to generate moDCs. Then, these moDCs were pulsed in vitro with various pancreatic islet autoantigens and then their “presentome” was analyzed (the eluted epitopes presented in the surface HLA-DR molecules) with mass spectrometry. Their experimental set up also held augmented clinical relevance as they selectively used moDCs from individuals possessing the alleles HLA-DR3 and HLA-DR4, associated with high-risk of disease emergence. Among their findings was the addition of new epitopes to those already characterized for some peptide autoantigens as well as the discovery of some derived from pancreatic islet peptides for which epitope generation had not been previously reported. Interestingly, they were able to show that not all the discovered epitopes induce a pro-inflammatory reaction, evident by the response magnitude and its induced IL-10 or IFN-α signature upon incubation of PBMCs from T1D patients with them. Importantly, the most immunodominant epitopes were generated by moDCs when compared to B cells, another immune system cell with antigen presentation capacity, in an HLA-DR allele independent manner. Thus, such approaches could help increase the efficacy of peptide-based tolerogenic immunotherapies as well as their patient-specific tailoring by using epitopes preferentially presented by each HLA haplotype in moDCs.

### 3.4. Bridging the Metabolic Profile and the Function of DCs in IMIDs: An Emerging Field of Research

The relevance of metabolomics in IMIDs is constantly increasing, however, its targeted application in DCs is only lately gaining attention. Towards that point, a recent study aimed to identify metabolic pathways exhibiting similar dysregulation in the circulation and DCs of patients with systemic sclerosis [160]. To this end, they first performed metabolite analysis using plasma of patients and healthy individuals and discovered evidence of imbalanced fatty acid and carnitine levels in systemic sclerosis samples. In line with this, they also found increased levels of L-carnitine in moDCs, derived from GM-CSF and IL-4 cultures of peripheral blood monocytes from systemic sclerosis patients, after their stimulation with TLR agonists. As a continuation of their observations, the authors tested the effect of etoposide, a carnitine transporter inhibitor widely used for cancer treatment, on the activation of patient-derived moDCs after TLR stimulation and showed secretion of reduced levels of pro-inflammatory cytokines such as IL-6 in its presence. As carnitine transports fatty acids into the mitochondria in order to facilitate their oxidation, the above observations suggest that targeted suppression of fatty acid oxidation in DCs could be helpful in decreasing the inflammation related to the particular IMID.

## 4. Conclusions, Challenges and Open Questions

Multi-omics data can play a crucial role in clinical practice in the near future, for predicting disease susceptibility, disease severity and treatment response or identifying new therapeutic targets for IMIDs. However, we are still in the dawn of this exciting new era. Building large-scale patient cohorts with high-quality clinical data consisting of patient demographics, disease response and multiple layers of omics data, as well as refined analytic approaches to handle these data, would contribute to a better understanding of mechanisms governing IMIDs biology and accelerate precision medicine.

Certain barriers need to be considered and overcome towards the vision of biomarker discovery and targeted new therapies for IMIDs. First of all, so far, high-throughput analysis is mostly restricted to total PBMCs of patients, with data extracted from diverse immune cell types being very limited. To analyze the genome, which is regarded as a stable feature for each individual, an easily accessible tissue, such as blood and analysis of whole PBMCs is broadly acceptable. However, many other types of omics, such as transcriptome, proteome and metabolome, vary between diverse immune cell types and tissues. Due to the high degree of complexity of the immune system, selective targeting of specific immune cell populations dictating the complex immune responses during IMIDs, such as DCs and Tregs, allows a deeper understanding of the mechanisms driving disease pathogenesis, with the prospect of identifying more precise therapeutic targets avoiding broad immunosuppression. Additional multi-omics data extracted from the analysis of Tregs and DCs specifically are needed to elucidate the degree of dysfunction rendering these cells pathogenic for IMIDs.

Secondly, the few existing studies utilizing diverse omics approaches to analyze DCs and Tregs in IMIDs are restricted to information extrapolated from a single omic level. A single omic data layer characterizes a specific biological process from one aspect. However, biological processes are based on interactions among genes, proteomes, metabolites, etc., and are regulated by epigenetic modifications. Single biomolecules or signaling pathways cannot fully explain biological mechanisms or functions. To acquire a comprehensive picture of the intrinsic molecular mechanisms driving disease pathogenesis, a systematic collection of multi-omics data is required. This increasing availability of multi-omic platforms and layers poses new challenges in data analysis. Integration and common visualization of multi-omics data are fundamental in comprehending connections across diverse molecular layers and in fully utilizing the multi-omics resources available to make breakthroughs in biomarker and therapy discovery. Artificial intelligence (AI) and machine learning (ML) approaches are techniques further required to identify and uncover clinically relevant biomarkers and biological processes that can be targeted for therapy. To achieve this vision, the interdisciplinary collaboration of biologists, computer scientists, mathematicians, and physicians is indispensable for the task of precision medicine, that holds the promise of clinically meaningful benefits for the individual patient with IMIDs.

Thirdly, the tissue (or source) where the immune cells to be studied are located is another critical aspect to be considered. Indeed, due to sample accessibility, fewer studies have been performed on tissues rather than blood. Taking into account: (a) the diversity of IMIDs, each manifesting into different tissue of the body; and (b) the recently appreciated residency of DCs and Tregs in non-lymphoid tissues such as skin, adipose tissue, lung, bone marrow, etc., with the ability to control local inflammatory responses and to express diverse transcriptional programs compared to peripheral blood or lymphoid organs, additional multi-omics studies investigating the role and function of distinct immune cells in diverse tissues are required, in order to acquire a more holistic view of the complexity of the mechanisms governing the development of IMIDs.

Finally, taking into consideration the dynamics, rapid responses and spatial particularity of the immune system, temporal and spatial omics studies will be meaningful in providing insights into the dynamic process dictating the manifestation of IMIDs. For example, the process of antigen uptake, presentation, immunological synapsis and cell-to-cell contact in the interplay of DCs and Tregs is highly dynamic and depends on the spatial position of immune cells, stroma and other non-immune counterparts. Despite its importance in the function of an immune response and immune-mediated diseases, our current knowledge is only basic, which calls for more extensive research.
